# Association of Perceived Threat, Negative Emotions, and Self-Efficacy With Mental Health and Personal Protective Behavior Among Chinese Pregnant Women During the COVID-19 Pandemic: Cross-sectional Survey Study

**DOI:** 10.2196/24053

**Published:** 2021-04-12

**Authors:** Phoenix Kit Han Mo, Vivian Wai In Fong, Bo Song, Jiangli Di, Qian Wang, Linhong Wang

**Affiliations:** 1 Center for Health Behaviours Research School of Public Health and Primary Care Chinese University of Hong Kong Hong Kong Hong Kong; 2 National Center for Women and Children's Health Chinese Center for Disease Control and Prevention Beijing China; 3 National Center for Chronic and Noncommunicable Disease Control and Prevention, Chinese Center for Disease Control and Prevention Beijing China

**Keywords:** COVID-19, pregnant women, depression, anxiety, self-efficacy, mental health, survey, threat, emotion

## Abstract

**Background:**

COVID-19 is an emerging infectious disease that has created health care challenges worldwide. Pregnant women are particularly affected by this disease.

**Objective:**

The aims of this study are to assess the levels of perceived threat (susceptibility, severity, impact), negative emotions (fear, worry), and self-efficacy of pregnant women in China related to COVID-19 and to examine their associations with mental health (depression and anxiety) and personal protective behavior (wearing a face mask).

**Methods:**

A total of 4087 pregnant women from China completed a cross-sectional web-based survey between March 3 and 10, 2020.

**Results:**

The prevalence of probable depression and anxiety was 48.7% (1989/4087) and 33.0% (1347/4087), respectively; 23.8% participants (974/4087) reported always wearing a face mask when going out. Of the 4087 participants, 32.1% (1313) and 36.4% (1490) perceived themselves or their family members to be susceptible to COVID-19 infection, respectively; 3216-3518 (78.7%-86.1%) agreed the disease would have various severe consequences. Additionally, 2275 of the 4087 participants (55.7%) showed self-efficacy in protecting themselves from contracting COVID-19, and 2232 (54.6%) showed efficacy in protecting their family members; 1303 (31.9%) reported a high level of fear of the disease, and 2780-3056 (68.0%-74.8%) expressed worry about various aspects of COVID-19. The results of the multivariate multinominal logistic regression analyses showed that perceived severity, perceived impact, fear, and worry were risk factors for probable depression and anxiety, while self-efficacy was a protective factor. The results of the multivariate logistic regression analysis showed that perceived susceptibility was associated with always wearing a face mask.

**Conclusions:**

Chinese pregnant women showed high levels of mental distress but low levels of personal protective behavior during the COVID-19 pandemic. Interventions are needed to promote the mental health and health behavior of pregnant women during the pandemic.

## Introduction

### COVID-19 as an Emerging Infectious Disease

In December 2019, a cluster of viral pneumonia cases of unknown cause, later named COVID-19, were detected in Wuhan, Hubei Province. After that, more cases of new infections and deaths were reported across cities in China. Due to the highly contagious nature of the disease, the COVID-19 outbreak spread worldwide in less than three months. As of February 1, 2021, a total of 102,339,513 confirmed cases of COVID-19 and 2,217,005 deaths were reported worldwide [1]. The outbreak of COVID-19 represents a public health emergency of international concern.

### Measures to Prevent the Spread of COVID-19 in China

Following the initial outbreak of COVID-19 in Wuhan, numerous measures were enacted to prevent further spread of the disease to other parts of China. On January 23, 2020, the Chinese government shut down Wuhan and two additional cities (Wenzhou and Shenzhen) by suspending all modes of transportation to and from these cities. Other measures included cancelling or postponing large public events (ie, Chinese New Year celebrations), prohibition of attendance at school and work, and closure of public amenities. These measures were part of the social distancing policies, which aimed to limit social contacts and human-to-human transmission [2]. Moreover, public information and education campaigns that encouraged personal protective behaviors were promoted through social media marketing and traditional media. A number of protective measures were also implemented to prevent the spread of COVID-19 among members of the workforce who wished to return to work [3]. These measures included enhancing the practice of hand hygiene and wearing a face mask, as well as organizational measures such as improvement of workplace hygiene; tracking the physical health status of employees; and dissemination of facts about COVID-19 prevention [3]. On February 2, 2020, the National Health Commission of China issued a new notice proposing a list of guidelines and recommendations for pregnant women and health care professionals in response to COVID-19. This notice urged strengthening of health counselling, screening, and follow-ups for pregnant women. Recommendations on personal protective measures, including social distancing, hand hygiene, and the use of a face mask when going out, were also stated in the notice [4].

### Possible Impact of COVID-19 on Pregnant Women

Due to the immunological changes that occur during pregnancy, pregnant women are generally susceptible to respiratory pathogens and the development of severe pneumonia. Therefore, it is believed that pregnant women may be more susceptible to COVID-19 [5]. Recent reviews found that the most commonly reported symptoms of COVID-19 in pregnant women were fever and dry cough, followed by fatigue, diarrhea, dyspnea, lymphocytopenia with elevated C-reactive protein, sore throat, and myalgia [6-8]. These clinical characteristics of COVID-19 are similar to those of nonpregnant adult patients [6-8]. More complications were observed in symptomatic pregnant women, including intensive care unit admissions, mechanical ventilation, and death [9,10]. Moreover, studies have shown that infected pregnant women are at significantly higher risk for caesarean delivery and preterm birth than the general pregnant population [6,7,11], which is associated with increased risk and complications in both mothers and neonates. A number of fetal and neonatal complications, such as stillbirth, neonatal death, low birth weight, fetal distress, thrombocytopenia accompanied by abnormal liver function, neonatal asphyxia, and death, were also reported [7,12]. Although some reviews reported a low risk of vertical transmission [8,13], it is important to note that most studies to date have found no conclusive evidence of vertical transmission of COVID-19 [6,7,10,11,14]. According to the World Health Organization, mothers with suspected or confirmed COVID-19 are recommended to initiate and continue breastfeeding and mother-infant contact because the benefits of breastfeeding and mother-infant contact substantially outweigh the potential risks of transmission of COVID-19 to the child [15]. As the situation of COVID-19 is constantly changing worldwide, understanding the effect of COVID-19 on pregnancy would be important and beneficial for the development of treatment and management to reduce the morbidity and complications of COVID-19 in both mothers and neonates. Very few studies to date have examined the perceptions, emotions, and mental and behavioral responses to COVID-19 of pregnant women in China.

### Mental Health and Personal Preventive Behavior During the COVID-19 Period

The uncertainty of COVID-19 has caused substantial psychological distress to the public. In studies among Chinese people, it was found that during the COVID-19 epidemic, 53.8% of respondents rated the psychological impact of the COVID-19 outbreak as moderate or severe [16], between 16.1% and 20.1% Chinese people scored higher than the cutoff for depression [12,17], and between 20.4% and 35.1% scored higher than the cutoff for anxiety [12,17,18]. Recent studies have revealed that females experience much greater levels of anxiety, depression, and stress than their male counterparts during the COVID-19 pandemic [19-21]. Studies have also shown that pregnant women experienced greater psychological distress than the general population during the COVID-19 epidemic [22,23], as they may face difficulties in accessing health care services due to suspension of transportation and other nonemergency services. Health services for pregnant women may also become limited as the health system is strained by the increasing number of infections. Furthermore, visiting clinics and hospitals for medical checkups may place pregnant women at increased risk of infection. Given the potential risk of COVID-19 infection in pregnancy, pregnant women may feel anxious visiting these facilities and worry about being infected during antenatal checkup. Furthermore, other studies that examined the effect of lockdown and quarantine on the mental health of pregnant population demonstrated a higher prevalence of symptoms of psychological distress (ie, anxiety, depression, posttraumatic stress symptoms, and insomnia [24,25]). As antenatal stress and mental health problems are associated with multiple adverse outcomes for the child [26,27], it would be pertinent to examine the prevalence and associated factors of mental distress among pregnant women during the COVID-19 period so that early interventions could be designed.

The virus of COVID-19 can be transmitted through respiratory droplets and contacts. From the experience of past epidemic outbreaks, personal protective behaviors such as wearing a mask are recommended to offer protections against virus infection. A recent study reported that the frequency of always wearing a face mask in the past week was 76.1%-85.5% among participants in Wuhan and Shanghai during the COVID-19 period [18]. Research has shown that countries with higher proportions of citizens that used face masks had fewer COVID-19 cases and controlled the epidemic much earlier than countries that discouraged the use of face masks [28]. The frequent use of face masks was found to be associated with a lower number of COVID-19 cases; the perceived benefits of wearing a face mask (eg, effectiveness in preventing virus spread from asymptomatic patients) also boosted confidence and reduced the risk of adverse mental health among Chinese people [28]. Studies among pregnant women during the severe acute respiratory syndrome (SARS) outbreak in Hong Kong found that approximately 70% wore a mask all or most of the time [29]. It was expected that the frequency of wearing a face mask among pregnant women would be high during the COVID-19 pandemic.

### Factors Related to Mental Health and Personal Protective Behavior

Cognitions are important determinants of health behaviors and mental health. Protection motivation theory is a prominent theory that highlights the important role of cognition in health [30]. Protection motivation theory posits that two parallel cognitive processes function to predict an individual’s motivation to perform a health behavior: threat appraisal and coping appraisal. Threat appraisal focuses on perceived susceptibility (ie, estimation of the possibility of harm from a threat) and perceived severity (ie, estimation of the degree of harm resulting from the threat), while coping appraisal focuses on self-efficacy (ie, level of confidence in coping with the threats) and other factors that may increase or decrease the adaptive response. The perception that the pandemic has caused a significant impact on one’s life may decrease the probability of the adaptive response. According to protection motivation theory, increased levels of threat and coping appraisal increase individuals’ protection motivation, leading to the performance of a health behavior. Protection motivation theory has been applied to COVID-19 preventive behavior among health care workers and the general public [31,32]. A meta-analysis also confirmed that increases in perceived susceptibility, perceived severity, and self‐efficacy facilitated adaptive intentions or behaviors [33]. 

Consistent with protection motivation theory, extensive studies have also demonstrated the important role of perceived threats on behavioral and psychological responses to a disease. A review by Bish and Michie [34] identified perceived susceptibility to the disease and perceived severity of the disease as important predictors of protective behaviors during a pandemic. A recent study revealed that individuals’ perceived severity of the COVID-19 outbreak is related to an increased level of mental health problems in the Chinese public [35]. Studies from other countries, such as the Philippines, Vietnam, and Turkey, also supported the findings that the perceived impact, perceived susceptibility, and perceived severity of COVID-19 are related to increased levels of mental health problems and preventive behaviors [20,21,36].

The literature has also suggested self-efficacy as an important determinant of mental health and behavior. Studies on epidemic outbreaks, such as SARS and H1N1, have found that self-efficacy was associated with better mental health [37] and higher levels of protective behaviors [38,39], while a lower level of self-efficacy was associated with higher fear related to the disease [40]. Studies during the COVID-19 epidemic also demonstrated that stronger self-confidence, stronger confidence in one’s health care providers, and having a good perception of one’s health status were significantly associated with lower risks of anxiety, depression, and stress as well as with a lower psychological impact of the pandemic [18,20].

The literature has also highlighted the importance of emotional factors in mental health and behavior. According to the appraisal tendency theory, emotions guide specific cognitive response or appraisal, leading to an effect on mental and behavioral outcomes [41]. Negative emotions, such as fear and worry, are associated with a tendency to perceive a situation as uncertain and less controllable, which prompts individuals to engage in precautionary behaviors. Furthermore, negative emotions may impair individuals’ cognitive processes in coping with stressful encounters [42], which drives them to perform a range of precautionary behaviors regardless of their scientific value. Supporting this notion, studies conducted during pandemics, such as H1N1 [43-45] and COVID-19 [36], have shown that negative emotions, such as fear of the pandemic, are associated with the practice of protective behaviors. Negative emotions related to epidemic outbreaks have also been consistently found to be detrimental to mental health [40,46]. It is therefore conjectured that during the COVID-19 pandemic period, pregnant women who showed negative emotions toward COVID-19 may have poorer mental health but be more likely to adopt protective behaviors.

### This Study

This study examined the prevalence and identified factors for depression, anxiety, and frequency of face mask wearing during the COVID-19 period among pregnant women in China. Based on the protection motivation theory and the appraisal tendency theory, the roles of perceived susceptibility, perceived severity, perceived impact of COVID-19, self-efficacy, and negative emotions (ie, fear and worry about COVID-19) were examined. It was hypothesized that perceived threat and negative emotions would be risk factors for depression and anxiety, while self-efficacy would be a protective factor. Furthermore, perceived threat, negative emotions, and self-efficacy were hypothesized to be protective factors of the frequency of wearing a face mask.

## Methods

### Participants

The target participants were pregnant women who were currently using health care services from maternal health care institutions in Mainland China. Pregnant women who intended to continue the pregnancy were eligible for the study, and those who planned to terminate their pregnancy were excluded.

### Procedure

A web-based cross-sectional survey was conducted from March 3-10, 2020. Participants were recruited from maternal health care centers in various provinces of China (ie, Beijing, Chongqing, Guangdong, Guangxi, Hainan, Shandong, Tianjin, and Xinjiang). These maternal health care centers provided antenatal health services to all pregnant women within the region, they contained a record and contact details of those women who were using their health services. Eligible women were first identified from the record and were invited to take part in the survey through WeChat. A quick response (QR) code and link to the web-based survey was provided; interested participants could directly access the web-based survey through scanning the QR code or clicking the link. Information about the purpose and procedure of the survey was provided on the first page of the web-based survey. Participants were assured that no direct identifiers (eg, name, email address, ID number) were collected, all data collected would remain confidential, and only the research team would have access to the data. Refusal to take part in the survey would not affect the services they would obtain at the health care center. Women who agreed to take part in the survey were asked to provide informed consent by clicking the “I agree” button before starting the web-based survey. The survey took 15-20 minutes to complete. Participants received no incentive for their participation. Ethical approval was obtained from the Survey and Behavioral Research Ethics Committee of The Chinese University of Hong Kong (SBRE-19-395). Approximately 5540 invitation messages were sent out, and a total of 4087 completed responses were collected (response rate: 73.8%).

### Measures

#### Sociodemographic Characteristics

Participants were asked to report their age, education level, and employment status.

#### Pregnancy-Related Characteristics

Participants were asked to report their parity, gestational age, and whether they had any pregnancy-related complications.

#### Perceived Susceptibility to COVID-19

The participants’ perceived susceptibility to COVID-19 was measured by 2 items on the likelihood of oneself and one’s family members contracting COVID-19. Items were rated on a 4-point Likert scale from 1, very little, to 4, very much, with a higher score indicating a higher level of perceived susceptibility. The internal reliability of the items was satisfactory (Cronbach α=.94).

#### Perceived Severity of COVID-19

The participants’ perception of the severity of COVID-19 was measured by 3 items (eg, “Would maternal infection with COVID-19 affect the health of the newborn?”). These items were rated on a 4-point Likert Scale from 1, very little, to 4, very much, with a higher score indicating a higher level of perceived severity. The internal reliability of the items was satisfactory (Cronbach α =.92).

#### Perceived Impact of COVID-19

Participants were given a checklist and were asked to rate whether COVID-19 had affected any part of their daily lives (ie, work, financial income, family relationship, social interactions, others). The number of items endorsed reflected the level of impact. The possible score ranged from 0 to 5, with a higher score indicating a higher level of perceived impact of COVID-19.

#### Self-efficacy

Self-efficacy was measured by 2 items. Participants were asked to rate their level of confidence of protecting themselves and their family members from contracting COVID-19. Items were rated on a 4-point Likert scale from 1, very little, to 4, very much, with a higher score indicating a higher level of self-efficacy. The internal reliability of the items was satisfactory (Cronbach α=.94).

#### Fear

Fear was measured by a single item. Participants were asked to rate their level of fear of COVID-19 on a 4-point Likert scale from 1, very little, to 4, very much, with a higher score indicating a higher level of fear.

#### Worry

Worry was measured by 4 items. Participants were asked to rate their level of worry regarding different aspects related to COVID-19 (eg, “You will be infected with COVID-19 when you attend the prenatal check-up”). Items were rated on a 4-point Likert scale from 1, very little, to 4, very much, with a higher score indicating a higher level of worry. The internal reliability of the items was satisfactory (Cronbach α=.92).

#### Frequency of Face Mask Wearing

Participants were asked to report their frequency of wearing a face mask when going out on a 4-point Likert scale from 1, never, to 4, always. A cutoff of always wearing a face mask was set in this study; this cutoff has been used in previous studies [44,47].

#### Depression

Depression was measured by the 9-item Patient Health Questionnaire (PHQ-9) [48]. The Chinese version has been validated and used in the Chinese population [49,50]. Participants were asked to rate how often they have been bothered by symptoms in the past 2 weeks on a 4-point Likert scale from 0, not at all, to 3, almost every day. The total score ranges from 0 to 27, with a higher score indicating a higher level of depression. Scores of 0-4, 5-9, 10-14, 15-19, and 20-27 represent minimal, mild, moderate, moderately severe, and severe depression, respectively.

#### Anxiety

Anxiety was assessed by the 7-item General Anxiety Disorder scale (GAD-7) [51]. It is a brief, self-reported scale for identifying probable cases of generalized anxiety disorder (GAD). The Chinese version has been validated and used in the Chinese population [52,53]. Participants were asked how often they experienced each symptom in the past 2 weeks on a 4-point Likert scale from 0, not at all, to 3, nearly every day. The GAD-7 total score ranges from 0 to 21, with a higher score indicating a higher level of anxiety. Scores of 0-4, 5-9, 10-14, and 15-21 represent minimal, mild, moderate, and severe anxiety, respectively.

### Analysis

Descriptive statistics on the participants’ sociodemographic and pregnancy-related characteristics, perceived threat, self-efficacy, negative emotions, and prevalence of depression, anxiety, and frequency of face mask wearing are presented. To identify significant factors of depression and anxiety, univariate multinominal logistic regressions were first conducted to examine the association between all factors with depression and anxiety, and respective odds ratios derived from univariate logistic regression (ORus) and 95% confidence intervals are presented. To control for the potential effect of sociodemographic and pregnancy-related characteristics, all sociodemographic characteristics, pregnancy-related characteristics, and independent variables with *P*<.05 in the univariate multinominal regression models were then subjected to multivariate multinominal logistic regression analysis; the resulting multivariate odds ratios (ORms) are reported.

To identify significant factors of always wearing a face mask when going out, univariate logistic regressions were first conducted to examine the association between all factors and the outcome, and the respective ORus and 95% confidence intervals are presented. To control for the potential effect of sociodemographic and pregnancy-related characteristics, all sociodemographic characteristics, pregnancy-related characteristics, and independent variables with *P*<.05 in the univariate logistic regressions were then subjected to a multivariate logistic regression analysis, and the resulting ORms were reported. Data analyses were performed using SPSS version 21.0 (IBM Corporation), with a *P* value of <.05 being considered statistically significant.

## Results

### Descriptive Statistics of the Participants

Slightly more than two-thirds (2743/4087, 67.1%) of the participants were aged 30 years or less. Nearly half of the participants (1989/4087, 48.7%) had received a postsecondary level of education. Approximately half of the participants (2022/4087, 49.5%) were nulliparous, and a similar number (1860/4087, 45.5%) were in their third trimester. A small number of participants (6.6%) reported having some pregnancy-related complications (Table 1).

**Table 1:** Characteristics of the participants (N=4087).

Characteristic				Value, n (%)
**Sociodemographic characteristics**				
	**Age (years)**			
		≥19	57 (1.4)	
		20-25	872 (21.3)	
		26-30	1814 (44.4)	
		31-35	1055 (25.8)	
		36-40	239 (5.8)	
		≥41	50 (1.3)	
	**Education level**			
		Primary or below	111 (2.7)	
		Junior secondary	1072 (26.2)	
		Senior secondary	915 (22.4)	
		Matriculation	1051 (25.7)	
		Undergraduate	831 (20.3)	
		Postgraduate or above	107 (2.6)	
**Pregnancy-related characteristics**				
	**Parity**			
		Nulliparous	2022 (49.5)	
		Primiparous	1820 (44.5)	
		Multiparous	245 (6.0)	
	**Gestational age**			
		First trimester (12 weeks or below)	855 (21.2)	
		Second trimester (13-26 weeks)	1362 (33.3)	
		Third trimester (27 weeks or above)	1860 (45.5)	
	**Pregnancy-related complications**			
		No	3816 (93.4)	
		Yes	271 (6.6)	
**Personal protective behavior**				
	**Frequency of wearing a face mask when going out**			
		Never	1262 (30.9)	
		Seldom	1398 (34.2)	
		Sometimes	453 (11.1)	
		Always	974 (23.8)	
**Mental health**				
	**Depression (measured by PHQ-9^a^)**			
		Minimal (0-4)	2098 (51.3)	
		Mild (5-9)	1163 (28.5)	
		Moderate (10-14)	504 (12.3)	
		Moderately severe (15-19)	227 (5.6)	
		Severe (20-27)	95 (2.3)	
	**Anxiety (measured by GAD-7^b^)**			
		Minimal (0-4)	2740 (67.0)	
		Mild (5-9)	919 (22.5)	
		Moderate (10-14)	318 (7.8)	
		Severe (15-21)	110 (2.7)	

^a^PHQ-9: 9-item Patient Health Questionnaire.

^b^GAD-7: 7-item General Anxiety Disorder Scale.

### Prevalence of Depression, Anxiety and Frequency of Wearing a Face Mask

The prevalence of mild to severe depression (PHQ score >5) and mild to severe anxiety (GAD-7 score >5) was 48.7% (1989/4087) and 33.0% (1347/4087), respectively. Less than a quarter of the participants (974/4087, 23.8%) reported always wearing a face mask when going out (Table 1).

### Perceived Threats, Negative Emotions, and Self-Efficacy

One-third of the 4087 participants perceived that they (1313, 32.1%) or their family members (1490, 36.4%) were likely to be infected with COVID-19 (ie, perceived susceptibility), and a sizable number of participants (3216, 78.7%, to 3518, 86.1%) agreed that the disease would have various severe consequences, such as mother-to-child transmission (ie, perceived severity; Table 2). Approximately one-fifth of respondents (894/4087, 21.9%) reported a score of 3 or above on perceived impact (Table 3). Slightly more than half were confident that they could protect themselves (2275/4087, 55.7%) or their family members (2232/4087, 54.6%) from contracting COVID-19. Approximately one-third (1303/4087, 31.9%) reported a high level of fear of the disease, and more than two thirds (2780/4087, 68.0%, to 3056/4087, 74.8%) showed worry about various aspects of COVID-19 (Table 2).

**Table 2:** Perceived threats, negative emotions, and self-efficacy of pregnant women during the COVID-19 period (N=4087).

Variable			Value, n (%)						
			Very little		Little		Much		Very much
**Perceived susceptibility**									
	Likelihood of oneself contracting COVID-19	788 (19.3)		1986 (48.6)		944 (23.1)		369 (9.0)	
	Likelihood of one’s family members contracting COVID-19	713 (17.4)		1884 (46.1)		1052 (25.7)		438 (10.7)	
**Perceived severity**									
	COVID-19 will be transmitted from mother to child	261 (6.4)		610 (14.9)		2114 (51.7)		1102 (27.0)	
	Maternal infection of COVID-19 will be more difficult to cure than in the general population	212 (5.2)		479 (11.7)		2074 (50.7)		1322 (32.3)	
	Maternal infection of COVID-19 will affect the health of the child	203 (5.0)		366 (9.0)		1944 (47.6)		1574 (38.5)	
**Self-efficacy**									
	Confidence of protecting oneself from contracting COVID-19	478 (11.7)		1334 (32.6)		1588 (38.9)		687 (16.8)	
	Confidence of protecting family members from contracting COVID-19	443 (10.8)		1412 (34.5)		1601 (39.2)		631 (15.4)	
**Fear**									
	Level of fear of COVID-19	666 (16.3)		2118 (51.8)		991 (24.2)		312 (7.6)	
**Worry**									
	Worry about being infected with COVID-19 when attending a prenatal checkup	258 (6.3)		773 (18.9)		1983 (48.5)		1073 (26.3)	
	Worry about one’s hospital delivery arrangement being affected due to COVID-19	371 (9.1)		936 (22.9)		1841 (45.0)		939 (23.0)	
	Worry that accompanied delivery will not be available due to COVID-19	398 (9.7)		865 (21.2)		1844 (45.1)		980 (24.0)	
	Worry that child health services will be affected after delivery due to COVID-19	339 (8.3)		746 (18.3)		1957 (47.9)		1045 (25.6)	

**Table 3:** Scores of perceived impact of COVID-19 (N=4087).

Total score of perceived impact	Value, n (%)
1	2138 (52.3)
2	1055 (25.8)
3	592 (14.5)
4-5	302 (7.4)

### Multinominal Logistic Regression Models for Depression

The results of the univariate multinominal logistic regression analyses showed that among all the background characteristics, being multiparous was a protective factor for mild depression (ORu 0.63, 95% CI 0.45-0.88) and being a farmer was a protective factor for moderately severe depression (ORu 0.72, 95% CI 0.53-0.96) (Table 4). Among the cognitive and psychological variables, perceived susceptibility (ORu 1.17-1.26), perceived severity (ORu 1.14-1.52) and fear (ORu 1.46-2.12) were risk factors for all levels of depression, and perceived impact was a risk factor for mild, moderate, and moderately severe depression (ORu 1.14-1.32). On the other hand, self-efficacy (ORu 0.89, 95% CI 0.82-0.96) was a significant protective factor for moderately severe depression (Table 4). The results of the multivariate multinominal regression analyses showed that after adjusting for significant background variables, perceived severity (ORm 1.08 and 1.09 for mild and moderate depression, respectively), perceived impact (ORm 1.08 and 1.12 for mild and moderate depression, respectively), fear (ORm 1.26-1.70 for all levels of depression), and worry (ORm 1.05 and 1.27 for moderate and severe depression, respectively) were significant risk factors, while self-efficacy was a protective factor (ORm 0.79-0.87 for moderate to severe depression) to different levels of depression (Table 5).

**Table 4:** Univariate multinominal logistic regression of depression among pregnant women (N=4087).

Variable			ORu^a^ (95% CI)			
			Mild depression	Moderate depression	Moderately severe depression	Severe depression
**Sociodemographic characteristics**						
	**Age (years)**					
		≤19^b^	1	1	1	1
		20-25	0.94 (0.50-1.77)	1.21 (0.49-3.00)	1.31 (0.39-4.44)	0.63 (0.18-2.21)
		26-30	1.08 (0.58-2.00)	1.21 (0.50-2.96)	0.88 (0.26-2.96)	0.36 (0.10-1.23)
		31-35	0.97 (0.52-1.81)	1.06 (0.43-2.61)	1.19 (0.35-4.02)	0.37 (0.10-1.31)
		36-40	1.05 (0.53-2.06)	1.14 (0.43-3.00)	0.79 (0.20-3.04)	0.47 (0.11-2.00)
		≥41	0.38 (0.14-1.07)	0.88 (0.26-3.03)	0.58 (0.09-3.75)	0.59 (0.09-3.76)
	**Education level**					
		Primary or below^b^	1	1	1	1
		Junior secondary	1.07 (0.65-1.76)	0.70 (0.40-1.23)	0.60 (0.17-2.11)	3.87 (0.52-28.70)
		Senior secondary	1.40 (0.85-2.30)	0.85 (0.48-1.50)	0.52 (0.23-1.19)	2.90 (0.38-21.91)
		Matriculation	1.54 (0.94-2.52)	0.83 (0.47-1.45)	0.73 (0.33-1.61)	2.17 (0.29-16.48)
		Undergraduate	1.61 (0.98-2.65)	0.81 (0.46-1.44)	0.52 (0.23-1.19)	1.87 (0.24-14.52)
		Postgraduate or above	1.90 (1.01-3.58)	0.87 (0.39-1.94)	0.60 (0.17-2.11)	2.40 (0.21-27.25)
	**Employment**					
		Unemployed/housewife/student^b^	1	1	1	1
		Farmer	1.01 (0.86-1.18)	0.96 (0.78-1.18)	0.72 (0.53-0.96)*	0.84 (0.54-1.30)
		Employed	0.87 (0.65-1.18)	0.98 (0.67-1.45)	1.16 (0.72-1.88)	0.99 (0.45-2.18)
**Pregnancy-related characteristics**						
	**Parity**					
		Nulliparous^b^	1	1	1	1
		Primiparous	0.90 (0.77-1.04)	0.87 (0.71-1.07)	1.30 (0.97-1.73)	1.30 (0.84-1.99)
		Multiparous	0.63 (0.45-0.88)**	0.89 (0.59-1.34)	1.59 (0.95-2.67)	1.31 (0.57-2.99)
	**Gestational age**					
		First trimester (12 weeks or below)^b^	1	1	1	1
		Second trimester (13-26 weeks)	1.00 (0.82-1.22)	0.95 (0.72-1.25)	0.94 (0.65-1.36)	0.75 (0.41-1.38)
		Third trimester (27 weeks or above)	0.95 (0.79-1.15)	1.15 (0.89-1.48)	0.82 (0.58-1.17)	1.18 (0.70-2.01)
	**Pregnancy-related complications**					
		No^b^	1	1	1	1
		Yes	1.12 (0.84-1.48)	1.02 (0.69-1.51)	1.03 (0.59-1.79)	0.81 (0.32-2.02)
**Cognitive and psychological variables**						
	Perceived susceptibility		1.17 (1.12-1.22)***	1.25 (1.18-1.32)***	1.26 (1.17-1.37)***	1.22 (1.09-1.38)**
	Perceived severity		1.14 (1.10-1.18)***	1.20 (1.15-1.26)***	1.22 (1.14-1.31)***	1.52 (1.34-1.72)***
	Perceived impact		1.14 (1.06-1.22)**	1.20 (1.09-1.33)***	1.32 (1.16-1.51)***	1.13 (0.92-1.39)
	Self-efficacy		1.01 (0.97-1.05)	0.96 (0.91-1.02)	0.89 (0.82-0.96)*	0.90 (0.80-1.01)
	Fear		1.46 (1.33-1.60)***	1.86 (1.65-2.10)***	2.10 (1.78-2.49)***	2.12 (1.66-2.71)***
	Worry		1.09 (1.07-1.12)	1.15 (1.12-1.19)	1.20 (1.15-1.26)	1.41 (1.29-1.53)

^a^ORu: odds ratio derived from univariate multinominal logistic regression.

^b^Reference category.

*P<.05.

**P<.01.

***P<.001.

**Table 5:** Multivariate multinominal logistic regression of depression among pregnant women (N=4087).

Variable	ORm^a^ (95% CI)			
	Mild depression	Moderate depression	Moderately severe depression	Severe depression
Perceived susceptibility	1.05 (0.99-1.12)	1.04 (0.96-1.12)	1.02 (0.92-1.13)	0.93 (0.81-1.07)
Perceived severity	1.09 (1.04-1.14)*	1.08 (1.01-1.15)**	1.02 (0.93-1.12)	1.14 (0.97-1.35)
Perceived impact	1.08 (1.00-1.17)**	1.12 (1.02-1.24)**	1.22 (1.07-1.40)	0.99 (0.81-1.23)
Self-efficacy	0.96 (0.92-1.00)	0.87 (0.82-0.93)*	0.79 (0.72-0.86)*	0.82 (0.72-0.93)**
Fear	1.26 (1.11-1.42)*	1.65 (1.40-1.94)*	1.91 (1.53, 2.40)*	1.70 (1.24-2.32)**
Worry	1.01 (0.98-1.05)	1.05 (1.00-1.10)**	1.11 (1.03-1.19)**	1.27 (1.13-1.43)*

^a^ORm: odds ratio derived from multivariate multinominal logistic regression that included all sociodemographic variables, pregnancy-related variables, and cognitive and psychological variables that were significant at the *P*<.05 level in the univariate multinominal logistic regression analysis.

*P<.001.

**P<.05.

### Multinominal Logistic Regression Models for Anxiety

Results from univariate multinominal logistic regressions showed that among all the background characteristics, being multiparous was a risk factor (ORu 2.51, 95% CI 1.32-4.75), while being a farmer was a protective factor (ORu 60, 95% CI 0.40-0.90) for severe anxiety. Education level (ORu 0.32 to 0.46 for matriculation and undergraduate level) was a protective factor for moderate anxiety. Among the cognitive and psychological variables, perceived susceptibility (ORu 1.20-1.24), perceived severity (ORu 1.19-1.52), fear (ORu 1.62-3.03), and worry (ORu 1.16-1.41) were risk factors for all levels of anxiety, and perceived impact was a risk factor for mild and moderate anxiety (ORu 1.16-1.29). On the other hand, self-efficacy (ORu 0.90-0.93) was a significant protective factor for moderate and severe anxiety (Table 6). The results from the multivariate multinominal regression analysis showed that after adjusting for significant background variables, fear (ORm 1.36-2.88) and worry (ORm 1.09-1.22) were risk factors, while self-efficacy was a protective factor (ORm 0.77-0.90) for all levels of anxiety. Furthermore, perceived severity (ORm 1.07, 95% CI 1.02-1.13) was a risk factor for mild anxiety, and perceived impact (ORm 1.22, 95% CI 1.09-1.37) was a risk factor for moderate anxiety (Table 7).

**Table 6:** Univariate multinominal logistic regression of anxiety among pregnant women (N=4087).

Variable				ORu^a^ (95% CI)		
			Mild anxiety		Moderate anxiety	Severe anxiety
**Sociodemographic characteristics**						
	**Age (years)**					
		≤19^b^	1		1	1
		20-25	0.57 (0.22-1.50)		0.99 (0.38-2.61)	0.59 (0.17-2.03)
		26-30	0.92 (0.47-1.80)		0.74 (0.29-1.93)	0.32 (0.10-1.10)
		31-35	0.75 (0.40-1.41)		0.76 (0.29-2.00)	0.53 (0.15-1.81)
		36-40	0.77 (0.41-1.42)		0.57 (0.19-1.70)	0.51 (0.13-2.07)
		≥41	0.72 (0.38-1.35)		0.57 (0.13-2.56)	0.63 (0.10-4.00)
	**Education level**					
		Primary or below^b^	1		1	1
		Junior secondary	0.99 (0.59-1.64)		0.58 (0.32-1.04)	5.25 (0.72-38.54)
		Senior secondary	1.21 (0.73-2.02)		0.69 (0.38-1.23)	2.45 (0.32-18.49)
		Matriculation	1.19 (0.72-1.98)		0.46 (0.25-0.83)*	2.05 (0.27-15.46)
		Undergraduate	1.31 (0.79-2.19)		0.32 (0.17-0.59)**	1.94 (0.25-14.91)
		Postgraduate or above	1.36 (0.70-2.63)		0.60 (0.25-1.44)	2.11 (0.19-23.87)
	**Employment**					
		Unemployed/housewife/student^b^	1		1	1
		Farmer	1.08 (0.92-1.27)		0.78 (0.61-1.00)	0.60 (0.40-0.90)*
		Employed	0.66 (0.93-0.68)		1.09 (0.71-1.68)	1.00 (0.51-1.97)
**Pregnancy-related characteristics**						
	**Parity**					
		Nulliparous^b^	1		1	1
		Primiparous	0.88 (0.76-1.03)		1.06 (0.84-1.35)	1.32 (0.88-1.98)
		Multiparous	0.86 (0.62-1.21)		1.37 (0.86-2.17)	2.51 (1.32-4.75)*
	**Gestational age**					
		First trimester (12 weeks or below)^b^	1		1	1
		Second trimester (13-26 weeks)	0.97 (0.78-1.20)		1.22 (0.88-1.70)	0.84 (0.48-1.48)
		Third trimester (27 weeks or above)	1.31 (1.07-1.60)*		1.21 (0.88-1.66)	1.36 (0.82-2.24)
	**Pregnancy-related complications**					
		No^b^	1		1	1
		Yes	1.39 (1.04-1.84)*		1.33 (0.86-2.06)	.90 (0.39-2.08)
**Cognitive and psychological variables**						
	Perceived susceptibility		1.20 (1.15-1.26)***		1.28 (1.20-1.37)***	1.24 (1.11-1.38)***
	Perceived severity		1.21 (1.16-1.25)***		1.19 (1.12-1.26)***	1.52 (1.35-1.71)***
	Perceived impact		1.16 (1.08-1.25)***		1.29 (1.15-1.44)***	1.15 (0.95-1.38)
	Self-efficacy		0.98 (0.94-1.02)		0.93 (0.87-0.99)*	0.90 (0.80-0.99)*
	Fear		1.62 (1.47-1.78)***		2.13 (1.85-2.45)***	3.03 (2.41-3.81)***
	Worry		1.16 (1.13-1.19)***		1.20 (1.15-1.25)***	1.41 (1.30-1.53)***

^a^ORu: Odds ratio derived from univariate multinominal logistic regression.

^b^Reference category.

*P<.05.

**P<.01.

***P<.001.

**Table 7:** Multivariate multinominal logistic regression of anxiety among pregnant women (N=4087).

Variable	ORm^a^ (95% CI)		
	Mild anxiety	Moderate anxiety	Severe anxiety
Perceived susceptibility	1.04 (0.98-1.10)	1.03 (0.94-1.13)	0.88 (0.74-1.02)
Perceived severity	1.07 (1.02-1.13)*	0.99 (0.91-1.07)	1.12 (0.96-1.31)
Perceived impact	1.07 (0.99-1.16)	1.22 (1.09-1.37)**	1.03 (0.85-1.24)
Self-efficacy	0.90 (0.86-0.95)***	0.83 (0.77-0.89)***	0.77 (0.68-0.87)***
Fear	1.36 (1.19-1.55)***	1.88 (1.55-2.29)***	2.88 (2.15-3.88)***
Worry	1.09 (1.05-1.13)***	1.12 (1.05-1.19)***	1.22 (1.09-1.37)***

^a^ORm: odds ratio derived from multivariate multinominal logistic regression that included all sociodemographic variables, pregnancy-related variables, and cognitive and psychological variables that were significant at the *P*<.05 level in the univariate multinominal logistic regression analysis.

*P<.05.

**P<.01.

***P<.001.

### Logistic Regression Models for Always Wearing a Face Mask When Going Out

Results from the univariate logistic regression analyses showed that among all the background characteristics, being in the third trimester (ORu 0.64, 95% CI 0.53-0.77) and pregnancy-related complications (ORu 0.69, 95% CI 0.50-0.95) were risk factors for always wearing a face mask when going out. Having a postgraduate level of education or above was a protective factor for always wearing a face mask (ORu 2.78, 95% CI 1.53-5.05) (Table 8). Among the cognitive and psychological variables, perceived susceptibility (ORu 1.07, 95% CI 1.02-1.11), perceived severity (ORu 1.05, 95% CI 1.02-1.09), and worry (ORu 1.03, 95% CI 1.00-1.05) were associated with always wearing a face mask. The results of the multivariate logistic regression analysis showed that after adjusting for significant background variables, perceived susceptibility was the only significant factor (ORm 1.05, 95% CI 1.00-1.10) for always wearing a face mask (Table 8).

**Table 8:** Logistic regression of always wearing a face mask when going out among pregnant women (N=4087).

Variable					Always wearing a face mask when going out		
			ORu^a^ (95% CI)			ORm^b^ (95% CI)	
**Sociodemographic characteristics**							
	**Age (years)**						
		≤19^c^	1			N/A^d^	
		20-25	1.36 (0.68-2.75)			N/A	
		26-30	1.61 (0.81-3.20)			N/A	
		31-35	1.32 (0.66-2.65)			N/A	
		36-40	1.51 (0.72-3.17)			N/A	
		≥41	2.42 (0.99-5.95)			N/A	
	**Education level**						
		Primary or below^c^	1			1	
		Junior secondary	1.98 (0.60-1.58)			0.92 (0.56-1.49)	
		Senior secondary	0.92 (0.57-1.50)			0.84 (0.51-1.39)	
		Matriculation	1.31 (0.81-2.12)			1.20 (0.73-1.98)	
		Undergraduate	1.57 (0.97-2.55)			1.45 (0.87-2.41)	
		Postgraduate or above	2.78 (1.53-5.05)***			2.43 (1.31-4.54)**	
	**Employment**						
		Unemployed/housewife/student^c^	1			1	
		Farmer	1.25 (1.06-1.46)*			0.99 (0.83-1.19)	
		Employed	0.88 (0.65-1.20)			0.91 (0.66-1.26)	
**Pregnancy-related characteristics**							
	**Parity**						
		Nulliparous^c^	1			N/A	
		Primiparous	0.86 (0.74-1.00)			N/A	
		Multiparous	0.99 (0.73-1.35)			N/A	
	**Gestational age**						
		First trimester (12 weeks or below)^c^	1			1	
		Second trimester (13-26 weeks)	0.86 (0.70-1.04)			0.90 (0.74-1.09)	
		Third trimester (27 weeks or above)	0.64 (0.53-0.77)***			0.68 (0.56-0.83)***	
	**Pregnancy-related complications**						
		No^c^	1			1	
		Yes	0.69 (0.50-0.95)*			0.70 (0.51-0.97)*	
**Cognitive and psychological variables**							
	Perceived susceptibility			1.07 (1.02-1.11)**			1.05 (1.00-1.10)*
	Perceived severity			1.05 (1.02-1.09)**			1.04 (.99, 1.09)
	Perceived impact			1.07 (1.00-1.15)			N/A
	Self-efficacy			1.00 (0.97-1.05)			N/A
	Fear			1.07 (0.98-1.17)			N/A
	Worry			1.03 (1.00-1.05)*			1.02 (0.98-1.05)

^a^ORu: odds ratio derived from univariate logistic regression.

^b^ORm: odds ratio derived from multivariate logistic regression that included all socio-demographic variables, pregnancy-related variables, and cognitive and psychological variables that were significant at the *P*<.05 level in the univariate logistic regression analysis.

^c^Reference variable.

^d^N/A: not applicable.

*P<.05.

**P<.01.

***P<.001.

## Discussion

### Principal Findings

The COVID-19 pandemic is an emerging and rapidly evolving situation. People receive information from various sources may form different perceptions about COVID-19. In this study, between 32.1% (1313/4087) and 36.4% (1490/4087) of the participants perceived that they or their family members were susceptible to the disease, and between 78.7% (3216/4087) and 86.1% (3518/4087) believed that the disease would have various serious consequences. Participants also reported that the disease has affected various parts of their lives. The perceived susceptibility and severity of COVID-19 were considerably higher compared to those observed in a study conducted among residents of Wuhan and Shanghai, which showed that 12.5%-18.6% of participants perceived that they were likely to be infected with COVID-19 and 12%-19.9% rated the disease as serious [18]. The figures were also higher compared with those in studies conducted during other epidemics, such as SARS (eg, approximately 20% of pregnant women considered themselves likely to contract SARS during the SARS epidemic period) [29]. Our findings suggest that a substantial number of pregnant women overestimated their risk of contracting COVID-19 and the severity of infection. Furthermore, slightly more than half of the participants reported high levels of self-efficacy. However, this figure was significantly lower than that in the study conducted in Wuhan and Shanghai, which showed that 86.8% to 87.8% of participants perceived a high level of confidence in taking measures to protect themselves from COVID-19 [18].

Negative emotions towards COVID-19 were commonly documented in this study. Approximately one-third of the 4087 participants (1303, 31.9%) showed high levels of fear towards the disease, and more than two thirds (2780, 68.0%, to 3056, 74.8%) reported different worries related to the COVID-19 pandemic, including worries of contracting the infection when attending antenatal checkups and worries that their delivery and child health services would be affected due to the epidemic. The level of negative emotions is comparable to that documented among pregnant women and the general public during the SARS period in Hong Kong, for which studies found that more than half of the participants worried about themselves or their family members contracting SARS and two-thirds of the women were scared of going to the hospital for antenatal visits [29,54]. Due to the high level of transmissibility of COVID-19 and the disruptions that the pandemic has caused in daily life, it is not surprising that high levels of fear and worry related to the disease were reported in this study. The psychological impact of an epidemic outbreak on pregnant women warrants immediate attention.

It is important to note that nearly half of this sample (1989/4087, 48.7%) scored above the cutoff for probable depression, and one-third (1347/4087, 33.0%) scored above the cutoff for probable anxiety. The prevalence was significantly higher than that reported in the general population of pregnant women [55] as well as that reported among the general public during the COVID-19 period [12,17,18]. These findings were also in line with those conducted in countries such as Canada and Japan, which also showed elevated depression and anxiety among pregnant women during the COVID-19 pandemic [56,57]. Our findings suggest that mental health problems were heightened among pregnant women during the COVID-19 pandemic. Furthermore, it is surprising that although a high level of perceived susceptibility, perceived severity, and perceived impact were reported, less than a quarter of the sample (974/4087, 23.8%) reported always wearing a mask when going out. The prevalence of wearing a face mask was substantially lower compared to that reported in studies among pregnant women and the general public during the SARS epidemic (ie, approximately 70%) [29,54], and the one reported in Wuhan and Shanghai during the COVID-19 pandemic [18]. Our findings show that pregnant women reported poor mental health and personal protective behavior during the COVID-19 epidemic. Efforts should be made to provide education to this specific at-risk group.

This study also identified some factors associated with mental health and protective behavior. The results of the multivariate analyses showed that among the cognitive variables, perceived severity and perceived impact of COVID-19 were associated with higher levels of depression and anxiety. These findings are in line with those of previous studies of epidemic outbreaks such as SARS, in which individuals who had a negative appraisal of the disease reported mental health problems [40,46]. Individuals who endorsed higher levels of negative impact of COVID-19 on their daily lives and perceived that they would experience serious consequences of the disease may have been more likely to exhibit negative emotional responses. Furthermore, perceived susceptibility was associated with higher likelihood of wearing a face mask when going out. These findings are consistent with the extant theories, such as protection motivation theory, that higher perceptions of risk and threat are associated with higher levels of engagement in health behaviors [33].

Consistent with previous studies that documented a positive association between self-efficacy and mental health outcomes [37,58], this study found that self-efficacy was associated with lower levels of depression and anxiety. Individuals with higher level of self-efficacy may have better ability to regulate the distress and negative mental health impact of the COVID-19 pandemic. They may be more likely to use more positive coping strategies to manage their emotions when encountering adversities associated with the disease. Surprisingly, despite the extensive evidence on the positive role of self-efficacy in explaining a range of health behaviors, self-efficacy had no significant association with wearing a face mask when going out in this study. In this study, self-efficacy was conceptualized as the perceived capability of protecting oneself or one’s family members from being infected with COVID-19; it was not behavior-specific in nature and may therefore have failed to predict face mask–wearing behavior.

Consistent with the literature that documented the negative impact of negative emotions and mental health [59,60], fear and worry were associated with higher levels of depression and anxiety in this study. However, no significant association between fear and worry with always wearing a mask when going out was found in this study. The findings suggest that the current sample of pregnant women may be more likely to regulate their negative emotions through mental health rather than behavioral response. More research is needed to elucidate the association between negative emotions and mental and behavioral outcomes under different contexts.

### Implications

The findings of this study provide implications for health care professionals and policy makers to address the threats posed to pregnant women by COVID-19 and by any possible future incidences of epidemic outbreaks. As poor health of pregnant women will lead to undesirable outcomes of both the mother and child, interventions to mitigate the negative effects of COVID-19 in this population is highly essential. First, this study found that cognitive perceptions on perceived threats of COVID-19 were associated with wearing a mask. To promote realistic risk perceptions and effective precautions of COVID-19, communication through various channels is essential. There is a need for governments and health care professionals to provide scientific information on the transmissibility and consequences of the disease and the efficacy of protective measures. However, because perceived threats are also associated with worse mental health outcomes, it is important to aim these health messages at increasing individuals’ threat-related beliefs while at the same time reducing misconceptions and adverse emotional and mental health outcomes.

Increasing self-efficacy should also be considered, as it has been found to be associated with better mental health. This increase can be achieved through cognitive restructuring of perceived capability in self-care and reappraisal of stressors related to the COVID-19 epidemic, as well as by providing encouragement and role modelling of positive behaviors. Digital psychotherapy, namely internet cognitive behavioral therapy (iCBT), has been demonstrated to be effective in combating the growing prevalence of mental health problems related to COVID-19 [61]. Previous studies have found that iCBT is efficacious in treating depression, anxiety, and insomnia [61,62]. In the context of COVID-19, iCBT could be a useful means for pregnant women to obtain mental health support without concern about contracting the disease from face-to-face contact [63]. As health care professionals are well-placed to recognize potential mental health problems at an early stage, routine screening for depression and other mental health conditions in pregnant women should be considered in obstetrical settings during the COVID-19 pandemic. Moreover, support from family, friends, and health care professionals can also promote the mental health of pregnant women by enhancing their perceived efficacy in coping with the disease.

Finally, the negative associations between negative emotions and mental health also suggest that health education should seek ways to disseminate a realistic level of risk that will not induce excessive worry and fear. It has also been shown that uncertainty can cause fear during pregnancy [64]. It is important to equip women with adequate knowledge about COVID-19 to reduce their fear and worry regarding the disease. Emotion regulation training can also be offered to improve their skills in regulating negative emotions.

### Limitations

The study was cross-sectional in nature, so causality cannot be assumed. Data were collected in a few cities; therefore, the findings may not be representative of the entire population of pregnant women in China. This study mainly used self-reported questionnaires to measure psychiatric symptoms and did not make clinical diagnoses. The standard for establishing a psychiatric diagnosis involves a structured clinical interview and functional neuroimaging [65,66]; therefore, the prevalence of mental health problems might have been overestimated. In addition, as participation was voluntary, the provinces that agreed to take part might have provided better services and support to pregnant women during the pandemic, leading to bias in the mental health and protective behaviors of the sample. Caution is needed when generalizing the findings to the population of pregnant women in China. In addition, the income levels of the pregnant women were not recorded. Lastly, as no validated scale on measuring perceptions of COVID-19 is available, variables were assessed by self-developed items with references to previous studies on other epidemics (eg, SARS), and some variables (ie, level of fear) were measured by a single item. The reliability of the items should be interpreted with caution.

### Conclusion

This study provided important insights on pregnant women’s perceptions of and emotional reactions to COVID-19 as well as their potential influence on behavioral and mental health. This study demonstrated that pregnant women reported a high level of perceived threat towards COVID-19; however, the frequency at which they wore a mask when going out was suboptimal. Most of the women showed negative emotions and mental responses. Our findings provide an important guide for health care professionals and policy makers to develop strategies to alleviate the negative effects of the COVID-19 epidemic.

## Abbreviations

GAD: generalized anxiety disorder
GAD-7: 7-item General Anxiety Disorder scale
iCBT: internet cognitive behavioral therapy
ORm: multivariate odds ratio
ORu: odds ratio derived from univariate logistic regression
PHQ-9: 9-item Patient Health Questionnaire
QR: quick response
SARS: severe acute respiratory syndrome
